# Identification of an H2-K^b^ or H2-D^b^ restricted and glypican-3-derived cytotoxic T-lymphocyte epitope peptide

**DOI:** 10.3892/ijo.2013.1793

**Published:** 2013-01-23

**Authors:** TATSUAKI IWAMA, KAZUTAKA HORIE, TOSHIAKI YOSHIKAWA, DAISUKE NOBUOKA, MANAMI SHIMOMURA, YU SAWADA, TETSUYA NAKATSURA

**Affiliations:** 1 Division of Cancer Immunotherapy, Research Center for Innovative Oncology, National Cancer Center Hospital East, Kashiwa, Chiba 277-8577;; 2 Research Institute for Biomedical Sciences, Tokyo University of Science, Japan

**Keywords:** glypican-3, cytotoxic T-lymphocyte epitope, hepatocellular carcinoma, mouse

## Abstract

Glypican-3 (GPC3) is overexpressed in human hepatocellular carcinoma (HCC) but not expressed in normal tissues except for placenta and fetal liver and therefore is an ideal target for cancer immunotherapy. In this study, we identified an H2-K^b^ or H2-D^b^ restricted and murine GPC3 (mGPC3)-derived cytotoxic T-lymphocyte (CTL) epitope peptide in C57BL/6 (B6) mice, which can be used in the design of preclinical studies of various therapies with GPC3-target immunotherapy *in vivo*. First, 11 types of 9- to 10-mer peptides predicted to bind with H2-K^b^ or H2-D^b^ were selected from the mGPC3 amino acid sequence based on the binding score as calculated by the BIMAS software. We evaluated the peptide-binding affinity and confirmed that all peptides were able to bind to H2-K^b^ or H2-D^b^ by *in vitro* cellular binding assay. Subsequently, a mixed peptide vaccine and single peptide vaccine were given to B6 mice to evaluate immunogenic potential of the 11 selected peptides. Using the splenocytes from peptide-vaccinated mice, interferon (IFN)-γ enzyme-linked immunospot (ELISPOT) assays showed that mGPC3-1_127–136_ (AMFKNNYPSL) peptide was the most efficient for inducing CTLs among the 11 peptides. Next, we demonstrated that the mGPC3-1 peptide-specific CTL line could recognize mGPC3-expressing cancer cells, suggesting that mGPC3-1 peptide was an endogenously presented peptide. In conclusion, we identified mGPC3-1 as an H2-K^b^ or H2-D^b^ restricted, mGPC3-derived CTL epitope peptide.

## Introduction

Liver cancer ranks fifth in frequency in the world and is the third most common cause of lethal cancer (1). Liver cancer consists of hepatocellular carcinoma (HCC) and intrahepatic cholangiocarcinoma (ICC), with HCC as the most common. Regarding HCC therapy, hepatectomy, percutaneous local therapy and transcatheter arterial embolization (TAE) are common, but the recurrence rate with conventional therapies for advanced HCC patients is still high (2). Therefore, developing a novel curative therapy or an effective adjuvant therapy for HCC is important.

Recently, immunotherapy, which consists of a peptide vaccine, protein vaccine, or DNA vaccine, has become a potentially promising option for HCC (3,4). Many tumor antigen-derived peptides recognized by cytotoxic T-lymphocyte (CTL) have been identified (5). However, to date, vaccine therapy using these peptides has not proven adequate antitumor efficacy in clinical trials for advanced HCC patients (6–8).

In HCC, glypican-3 (GPC3) is overexpressed and is not expressed in normal tissues except for the placenta and embryonic liver (9). Hence, GPC3 is a novel target molecule in HCC patients. GPC3 is a member of the heparan sulfate proteoglycan family and the glypican family regulates cell growth and division through Wnt signaling, Hedgehogs, fibroblast growth factors and bone morphogenetic proteins (10–12). We previously identified HLA-A^*^24:02-restricted GPC3_298–306_ (EYILSLEEL) and HLA-A*02:01-restricted GPC3_144–152_ (FVGEFFTDV) peptides and showed that both peptides can induce GPC3-specific CTLs without an autoimmune response (13,14). Clinical trials of a GPC3-derived peptide vaccine for HCC patients are currently in progress. The phase I clinical trial of a GPC3-derived peptide vaccine for advanced HCC showed safety as well as immunological evidence and potential for improving overall survival (15–17). The phase I clinical trial suggested that the GPC3-derived peptide vaccine could be an attractive approach for treatment of HCC, however, the effect of tumor reduction was limited. Therefore, further studies are needed to enhance the effect of GPC3-targeted immunotherapy and to establish a GPC3-specific CTL-inducible mouse model. We previously conducted a preclinical study of the GPC3-derived peptide vaccine using HLA-A2.1 transgenic mice (18). The treatment model experiment using HLA transgenic mice is limited.

Mice with the C57BL/6 (B6) background have been reported to spontaneously develop liver cancer (19,20). Recently, the NASH mouse model (named STAM mice C57BL/6N-NASH), which had a B6 background and spontaneously developed liver cancer, was exploited by Stelic Institute & Co. In this mouse model, the cancer incidence rate is high and cancer incident time is short, thus, STAM mice C57BL/6N-NASH is an attractive model for studying GPC3-targeted therapy for HCC. Therefore, identification of a mouse major histocompatibility complex (MHC) class I epitope peptide to induce GPC3-specific CTL was needed for establishment of the appropriate mouse model.

Strategies to identify epitope peptides have previously been reported (21–24). A summary of our strategy follows. First, peptides binding MHC class I epitope were predicted from antigen amino acid sequences *in silico* by prediction software and the ability of the predicted peptides to bind MHC class I was confirmed *in vitro* by a binding assay. Then, the immunogenic potential of the predicted peptides was examined by *in vivo* immunization or *in vitro* stimulation. Lastly, whether peptides that have immunogenic potential are presented by cells endogenously expressing the antigen was confirmed. In summary, we identified peptides with immunogenic potential that were presented by cells endogenously expressing the antigen. We attempted to identify H2-K^b^ or H2-D^b^ restricted, GPC3-derived CTL epitope peptides in C57BL/6 mice based on the above strategy.

## Materials and methods

### Mice

C57BL/6 (B6) mice were purchased from Charles River Laboratories Japan, Inc. and STAM mice C57BL/6N-NASH were a gift from this company. Mice were maintained under the institutional guidelines set by the Animal Research Committee of the National Cancer Center Hospital East. Mice were housed in specific pathogen-free (SPF) conditions with a 12-h light cycle and food and water at *ad libitum*. Six to eight-week-old female B6 mice were used in all experiments and STAM mice C57BL/6N-NASH were provided with a very high-fat rodent diet (rodent diet with 60% kcal% fat, Research Diet Inc.). All animal procedures were performed according to the guidelines for Animal Research Committee of the National Cancer Center, Japan.

### Cell lines and transfection

B6 thymoma RMA and RMA-S cell lines, which have H2-K^b^ and -D^b^ as MHC class I epitopes, were maintained in our laboratory. RMA-S is an antigen processing-defective cell line and the cells cannot present endogenous antigens with MHC class I epitopes (25). To obtain RMA transiently expressing murine GPC3 (RMA-GPC3-puro), RMA (GPC3-negative) was transfected with pCAGGS-mGPC3-internal ribosomal entry site (IRES)-puromycin-resistant (puro-R) using Lipofectamine 2000 reagent (Invitrogen Corp., Carlsbad, CA, USA) according to the manufacturer’s protocols. As negative control, RMA, which was transfected with pCAGGS-IRES-puro-R in a similar way, was named RMA-puro. Expression of murine GPC3 (mGPC3) in RMA-GPC3-puro or RMA-puro was confirmed by reverse transcription polymerase chain reaction (RT-PCR). All cells were cultured in RPMI-1640 (Gibco, USA) supplemented with 10% fetal bovine serum (FBS) (Gibco).

### RT-PCR

Total ribonucleic acid was isolated from RMA-GPC3-puro or RMA-puro homogenized with the TRIzol Reagent (Life Technologies, Inc., Rockville, MD, USA) according to the manufacturer’s protocols. The first-strand complementary deoxyribonucleic acid (cDNA) was synthesized with a PrimeScript^®^ II 1st strand cDNA Synthesis kit (Takara Bio Inc., Japan), then mGPC3 was amplified using a Takara PCR Amplification kit (Takara Bio Inc.). The amplification protocol was as follows: 150 sec at 94°C for initial denaturation, 35 amplification cycles at 58°C for 40 sec and 72°C for 40 sec, followed by a final extension at 72°C for 5 min. The primer sequences for mGPC3 were as follows: sense, 5′-ACGGGATGGTGAAA GTGAAGA-3′ and antisense, 5′-GAAAGAGAAAAGAGGGA AACA-3′. The primer sequences for β-actin were as follows: sense, 5′-GAGCAATGATCTTGATCTTCAT-3′ and antisense, 5′-TCCATCATGAACTGTGACGT-3′. PCR products were visualized by ethidium bromide staining after separation on a 1% agarose gel. After normalization using β-actin messenger ribonucleic acid (mRNA) as a control, we compared the expression of mGPC3 mRNA.

### Generation of bone marrow-derived dendritic cells (BM-DCs) from BM cells

BM cells (4×10^6^) from B6 mice were cultured in RPMI-1640 containing FBS (10%), 2-mercaptoethanol (2-ME, 50 *μ*M) and murine granulocyte macrophage colony-stimulating factor (mGM-CSF, 20 ng/ml) for 1 week.

### Peptides

Eleven types of 9- to 10-mer peptides predicted to bind with H2-K^b^ or H2-D^b^ were selected from mGPC3 amino acid sequences (accession code AAH36126) based on the binding score as calculated by BIMAS software (BioInformatics and Molecular Analysis Section, Center for Information Technology, NIH, Bethesda, MD, USA) and 11 synthetic peptides (custom ordered) were purchased from Scrum Inc. (Tables I and II). The 11 amino acid sequences were as follows: mGPC3-1, AMFKNNYPSL; mGPC3-2, SLFPVIYTQM; mGPC3-3, LFPVIYTQM; mGPC3-4, KSFINFYSAL; mGPC3-5, LTARLNMEQL; mGPC3-6, LGSDINVDDM; mGPC3-7, QYVQKNGGKL; mGPC3-8, YVQKNGGKL; mGPC3-9, DTLCWNGQEL; mGPC3-10, RNGMKNQFNL; mGPC3-11, MKNQFNLHEL. Each peptide was dissolved in dimethyl sulfoxide (DMSO) (Wako Pure Chemical Industries, Japan) and each peptide’s density was 10 mg/ml.

### H2-K^b^ or H2-D^b^ binding assay

To evaluate the binding affinity of the predicted peptides to H2-K^b^ or H2-D^b^ molecules, an *in vitro* cellular binding assay was performed as previously reported (23,26). Briefly, after incubation of RMA-S cells in culture medium at 26°C overnight, cells (1×10^6^) were washed with PBS and suspended in 100 *μ*l Opti-MEM^®^ (Invitrogen) with or without 10 *μ*g peptide, followed by incubation at 26°C for 3 h and then at 37°C for 3 h. After washing with PBS, H2-K^b^ or H2-D^b^ expression was measured with a BD FACSCanto™ II flow cytometer (BD) using FITC-conjugated H2-K^b^ (BioLegend Inc., AF6-88.5) or H2-D^b^ (BioLegend Inc., KH95) specific monoclonal antibody and mean fluorescence intensity (MFI) was recorded. Percent MFI increase was calculated as follows: percent MFI increase = (MFI with the given peptide - MFI without peptide)/(MFI without peptide) × 100.

### Vaccination

The mixed peptide vaccine per mouse consisted of 5 *μ*l mGPC3-1 to mGPC3-11 solution, 55 *μ*l sodium bicarbonate solution and 110 *μ*l incomplete Freund’s adjuvant (IFA). Single peptide vaccine per mouse consisted of 5 *μ*l peptide, 45 *μ*l sodium bicarbonate solution and 50 *μ*l IFA. Each vaccine solution was emulsified. The mice were immunized by intradermal injection at the base of the tail every 7 days for a total of two vaccinations. Similarly, STAM mice C57BL/6N-NASH were immunized seven times with the mGPC3-1 peptide vaccine.

### Restimulation of splenocytes obtained from immunized mice

Seven days after the last immunization, splenocytes were collected and cluster of differentiation 8 (CD8) positive splenocytes were isolated by positive selection with anti-CD8 microbeads (Miltenyi Biotec) according to the manufacturer’s protocol. CD8-positive splenocytes were cocultured with BM-DCs pulsed with each peptide as previously described (13). Seven days after coculture, the detection of antigen-specific T cells producing interferon (IFN)-γ was performed using the BD ELISPOT kit (BD Bioscience, San Jose, CA, USA) according to the manufacturer’s protocols.

### Establishment of GPC3-1-specific CTL line

The GPC3-1-specific CTL line was established as previously described (27). Splenocytes (1×10^4^) derived from B6 mice immunized with the GPC3-1 peptide vaccine were cocultured with B6-derived and irradiated (35 Gy) splenocytes (5×10^4^) in RPMI-1640 contained with FBS (10%), sodium pyruvate (1 mM, Gibco), MEM non-essential amino acid solution (1X, Gibco) and 2-ME (50 *μ*M). Seven days later, recombinant interleukin-2 (rIL-2, 50 U/ml, Nipro, Osaka, Japan) was added to the culture medium.

### IFN-γ enzyme-linked immunospot (ELISPOT) analysis

IFN-γ ELISPOT assay was performed according to the manufacturer’s protocols. Briefly, restimulated CD8-positive splenocytes (5×10^4^) as target cells were added to the plate and then BM-DCs (5×10^4^) pulsed with each peptide (10 *μ*g/ml) as effector cells or non-pulsed BM-DCs (5×10^4^) as control and target cells were added to the plate, which was then incubated for 20 h at 37°C, 5% CO_2_. Using the GPC3-1-reactive CTL line (1×10^5^) as effector cells, RMA-S (5×10^4^) pulsed with each peptide (10 *μ*g/ml) as target cells and non-pulsed RMA-S as control and target cells (5×10^4^), the plate was incubated for 20 h at 37°C, 5% CO_2_. Using the mGPC3-1-reactive CTL line (1×10^5^) as effector cells, RMA-GPC3-puro as target cells (5×10^5^) and RMA-puro (5×10^5^) as control and target cells, the plate was incubated for 48 h at 37°C, 5% CO_2_. The number of spots was automatically counted using the Eliphoto system (Minerva Tech, Tokyo, Japan).

### Cytotoxicity assay

Cytotoxic activity against target cells was analyzed using the Terascan VPC system (Minerva Tech) as previously described (28). Target cells were incubated with calcein AM (Dojindo, Kumamoto, Japan) solution for 30 min at 37°C and labeled. Then the labeled cells were incubated with effector cells for 4 h. Fluorescence intensity was measured before and after the culture and specific cytotoxic activity was evaluated using the following formula: % cytotoxicity = {1- [(average fluorescence of the sample wells - average fluorescence of the maximal release control wells) - (average fluorescence of the minimal release control wells - average fluorescence of the maximal release control wells)]} × 100%.

### Statistical analysis

Statistical analyses were performed with a Mann-Whitney U test (n=3). Significant differences were defined as ^*^p<0.05 or R^2^ >0.5.

## Results

### Evaluation of selected peptide-binding affinity to H2-K^b^ or H2-D^b^

**Figure 1:**
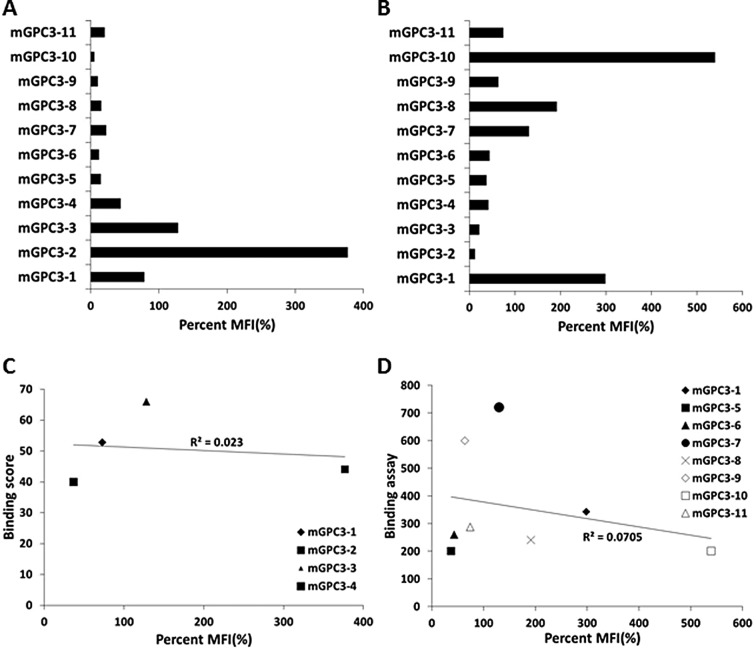
*In vitro* cellular peptide binding assays to H2-K^b^ (A) or H2-D^b^ (B) were performed using a FACS system. Comparison of BIMAS binding score with percent MFI for H2-K^b^ (C) or H2-D^b^ (D). Percent MFI increase = (MFI with the given peptide - MFI without peptide)/(MFI without peptide) × 100.

The selected 11 peptides derived from mGPC3 by the BIMAS software were evaluated by an *in vitro* binding assay to determine each peptide’s binding affinity to H2-K^b^ or H2-D^b^. The peptide with the highest binding affinity for H2-K^b^ was mGPC3-2 (percent MFI, 376.6%), followed by the mGPC3-3 peptide (128.0%) and the mGPC3-1 peptide (72.7%) (Fig. 1A). That for H2-D^b^ was mGPC3-10 peptide (539.1%) followed by the mGPC3-1 peptide (298.2%) and the mGPC3-8 peptide (191.1%) (Fig. 1B). These results show that all 11 peptides could bind H2-K^b^ or H2-D^b^, although the binding score calculated by the BIMAS software did not correlate with the actual binding affinity (Fig. 1C and D).

### Induction of CTL response against mGPC3-derived peptides in B6 mice

**Figure 2:**
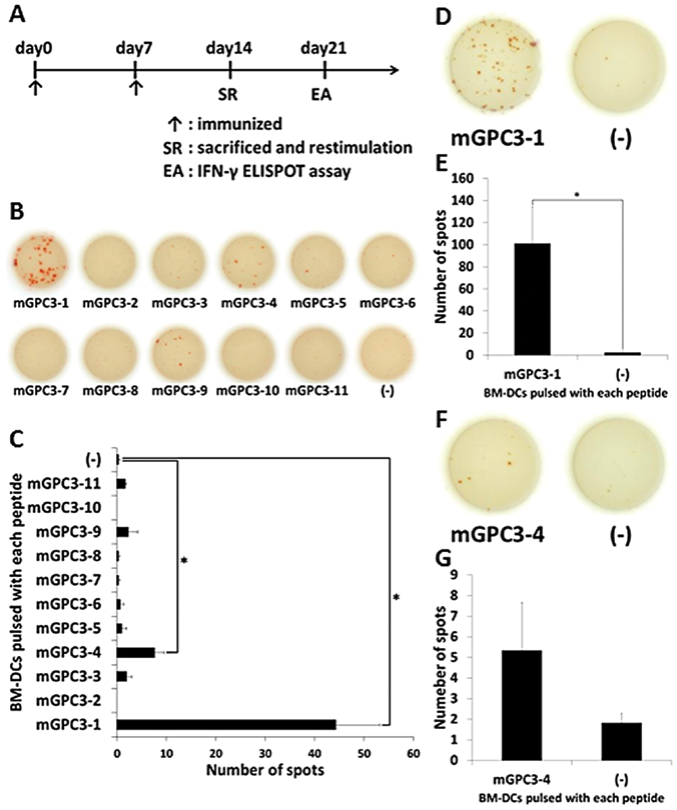
*In vivo* mixed peptide vaccine and single peptide vaccine. Analysis was performed for each vaccine. (A) Schedule of mixed peptide vaccine and single peptide vaccine. (B and C) The mixed peptide vaccine was given to mice and the responses of CD8-positive cells to the 11 peptides were examined. IFN-γ ELISPOT assays were performed using BM-DCs pulsed with each peptide and non-pulsed BM-DCs as target cells (n=3, *p<0.05). Representative data are shown (B). To confirm whether mGPC3-1 or mGPC3-4 was a CTL-inducible peptide, the single peptide vaccine was given. (D and E) mGPC3-1 peptide vaccine was given and IFN-γ ELISPOT assays were performed using BM-DCs pulsed with mGPC3-1 and non-pulsed BM-DCs as target cells (n=3, *p<0.05). Representative data are shown D). (F and G) mGPC3-4 peptide vaccine was given and IFN-γ ELISPOT assays were performed using BM-DCs pulsed with mGPC3-4 and non-pulsed BM-DCs as target cells (n=3). Representative data are shown (F).

The vaccine schedule was performed as follows (Fig. 2A): At days 0 and 7, peptide vaccine was given. At day 14, primed mice were sacrificed and CD8-positive splenocytes were collected. CD8-positive splenocytes were restimulated with BM-DCs pulsed with each peptide. At day 21, the peptide’s immunogenic potential was evaluated by IFN-γ ELISPOT assay.

The mixed peptide vaccination was performed to evaluate immunogenic potential of the 11 peptides and IFN-γ ELISPOT assays were performed using BM-DCs pulsed with each peptide and non-pulsed BM-DCs as target cells. The CD8-positive splenocytes from mice primed with the mixed vaccine released more IFN-γ to BM-DCs pulsed with mGPC3-1 peptide (average number of spots, 44.3±15.3) and mGPC3-4 peptide (average number of spots, 7.6±3.2) than to non-pulsed BM-DCs (average number of spots, 0.3±0.5). These results suggest that the mGPC3-1 and mGPC3-4 peptides had immunogenic potential and were able to induce peptide-specific CTLs in B6 mice primed by the mixed vaccine system (Fig. 2B and C).

Next, to confirm whether the peptides are CTL-inducible peptides, a single peptide vaccine was given and IFN-γ ELISPOT assays were performed using BM-DCs pulsed with either peptide and non-pulsed BM-DCs as target cells. The CD8-positive cells from mice immunized with mGPC3-1 peptide released more IFN-γ to BM-DCs pulsed with mGPC3-1 peptide (average number of spots, 101.0±33.2) than to non-pulsed BM-DCs (average number of spots, 2.1±3.7) (Fig. 2D and E). The CD8-positive cells from mice immunized with mGPC3-4 peptide released more IFN-γ to BM-DCs pulsed with mGPC3-4 peptide (average number of spots, 5.3±4.0) than to non-pulsed BM-DCs (average number of spots, 1.8±0.7), but no significant differences were observed (Fig. 2F and G). These results suggest that mGPC3-1 peptide is more efficient for inducing CTLs than the mGPC3-4 peptide in a single peptide vaccine system.

Taken together, the above results suggest that mGPC3-1 peptide is the most efficient peptide for inducing CTLs among the 11 peptides.

### mGPC3-1 peptide-specific CTL line recognition of target cells endogenously expressing mGPC3

**Figure 3:**
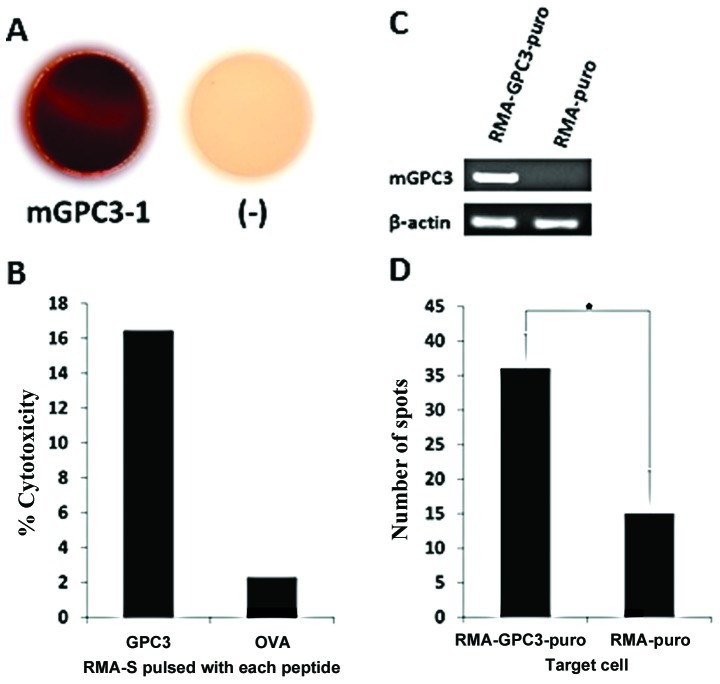
Analysis of established CTL line. (A) IFN-γ ELISPOT assays were performed using GPC3-1 pulsed or non-pulsed RMA-S as target cells. (B) Cytotoxicity assays were performed using GPC3-1 pulsed or unpulsed RMA-S as target cells. Percent cytotoxicity = {1- [(average fluorescence of the sample wells - average fluorescence of the maximal release control wells) - (average fluorescence of the minimal release control wells - average fluorescence of the maximal release control wells)]} × 100%. (C) mGPC3 expression of RMA-GPC3-puro and RMA-GPC3 by RT-PCR. (D) IFN-γ ELISPOT assays were performed using RMA-GPC3-puro and RMA-puro as target cells (n=3, ^*^p>0.05).

To further investigate the ability of mGPC3-1 peptide-specific CTLs induced by peptide vaccination, we established a CTL line from immunized mice according to the above described protocol. IFN-γ ELISPOT assays were performed using RMA-S pulsed with mGPC3-1 peptide and non-pulsed RMA-S to confirm whether the CTL line had mGPC3-1 peptide specificity. The CTL line clearly released more IFN-γ to RMA-S pulsed with mGPC3-1 peptide than to non-pulsed RMA-S, which suggests that the CTL line is the mGPC3-1 peptide-specific CTL (Fig. 3A).

Subsequently, a cytotoxicity assay was performed to confirm whether the mGPC3-1-specific CTLs could kill RMA-S pulsed with mGPC3-1 peptide. The CTLs killed RMA-S pulsed with the mGPC3-1 peptide (16.4%) better than non-pulsed RMA-S (2.2%), suggesting that the mGPC3-1-specific CTL line could specifically recognize and kill RMA-S pulsed with the mGPC3-1 peptide (Fig. 3B).

Finally, we examined whether the CTL line could recognize RMA GPC3-puro endogenously expressing mGPC3. Expression of mGPC3 in RMA-GPC3-puro and RMA-puro was confirmed by RT-PCR. The results showed that RMA-GPC3-puro expressed mGPC3 and RMA-puro did not express mGPC3 (Fig. 3C). IFN-γ ELISPOT assays were performed using RMA-GPC3-puro and RMA-puro as target cells to investigate whether the CTL line could recognize RMA-GPC3-puro expressing endogenous mGPC3. The CTL line released more IFN-γ to RMA-GPC3-puro (average number of spots, 32.2±5.0) than to RMA-puro (average number of spots, 18.2±6.2). This result suggests that the mGPC3-1 peptide is an endogenously presented peptide (Fig. 3D).

### CTL response against the mGPC3-derived peptides induced in STAM mice

**Figure 4:**
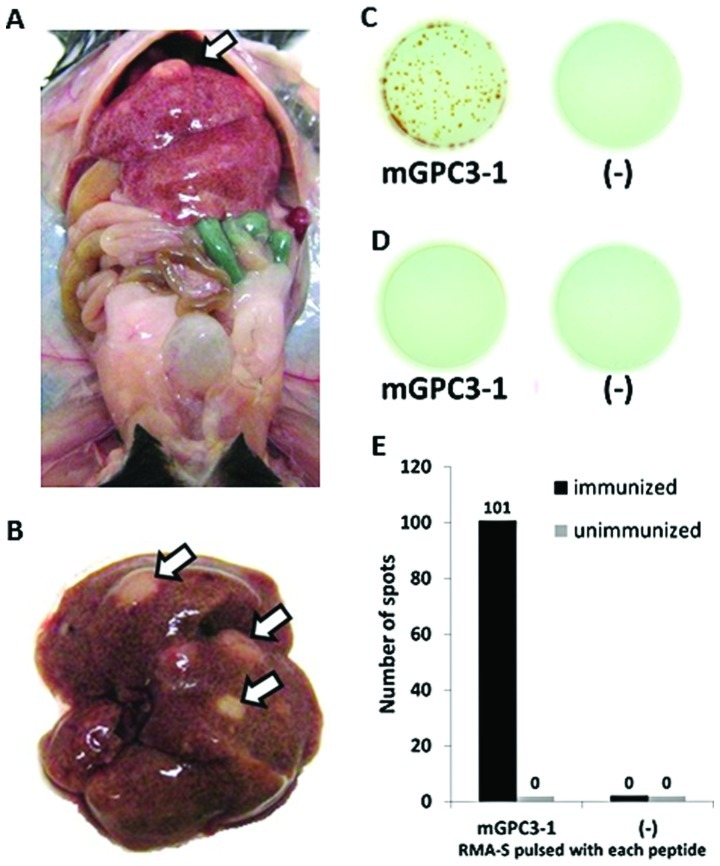
Analysis of STAM mice C57BL/6N-NASH. (A and B) Liver cancer was observed in 18-week-old STAM mice C57BL/6N-NASH. (C–E) To confirm whether mGPC3-1 peptide-specific CTL was induced, the mGPC3-1 peptide vaccine was given to STAM mice C57BL/6N-NASH and the IFN-γ ELISPOT assay was performed. Arrow indicates the area of developing cancer (C and E). The CD8-positive cells derived from immunized mice released IFN-γ to RMA-S pulsed with mGPC3-1 peptide (n=3). Representative data are shown (C). (D and E) As a control, IFN-γ ELISPOT assays were performed using the CD8-positive cells derived from unprimed mice. Representative data are shown (D).

Previously, the NASH mouse model (named STAM mice C57BL/6N-NASH) was exploited by Stelic Institute & Co. and STAM mice with a B6 background spontaneously developed liver cancer. We observed that liver cancer developed in 18-week-old STAM mice (Fig. 4A and B). Furthermore, to verify whether mGPC3-1 peptide-specific CTLs were induced in STAM mice C57BL/6N-NASH, a mGPC3-1 peptide vaccine was given and an IFN-γ ELISPOT assay was performed using RMA-S pulsed with mGPC3-1 peptide or non-pulsed RMA-S. The CD8-positive cells derived from immunized mice released IFN-γ only to pulsed RMA-S (average number of spots, 100±74.3), not to non-pulsed RMA-S (average number of spots, 0.0±0.0) (Fig. 4C and E). However, the CD8-positive cells derived from unimmunized mice did not release IFN-γ to either pulsed (average number of spots, 0±0.0 or non-pulsed (average number of spots, 0.0±0.0) RMA-S (Fig. 4D and E). These results suggest that peptide-specific mGPC3-1 could be induced in STAM mice C57BL/6N-NASH immunized with the mGPC3-1 peptide vaccine but could not be induced in un-immunized STAM mice C57BL/6N-NASH.

## Discussion

HCC is the most common liver cancer and the recurrence rate for treated HCC patients is high, thus establishment of an effective preventative method, such as a vaccination to prevent the occurrence and recurrence of HCC, is needed. GPC3 is overexpressed in HCC and is not expressed in normal tissue except for the placenta and embryonic liver. Clinical trials of a GPC3-derived peptide vaccine for HCC have been performed and a phase I clinical trial has shown the safety and immunological and clinical potential of the vaccine (15,16). Moreover, to study the preventive effect as a potential of the GPC3-derived peptide vaccine, we attempted to establish a mouse model to induce GPC3-specific CTLs by the peptide vaccine.

First, mGPC3-derived peptides binding to H2-K^b^ or H2-D^b^ were determined *in silico* using BIMAS software. Moreover, a binding assay was performed *in vitro* and showed that all peptides predicted by the BIMAS software could bind H2-K^b^ and H2-D^b^. However, the BIMAS score did not correlate with the actual binding affinity.

Peptides that can bind to MHC class I are not always able to induce peptide-specific CTLs (21,29). Therefore, to investigate actual CTL-inducible peptides among the 11 selected peptides, a mixed peptide vaccine and single peptide vaccine were given to mice. These results (Fig. 2) suggested that mGPC3-1 could induce peptide-specific CTLs. In addition, antigen-derived and CTL-inducible peptides are not necessarily presented by cancer cells endogenously expressing the antigen (23,30). Hence, we confirmed whether the mGPC3-1 peptide-specific CTL line could recognize RMA-GPC3-puro endogenously expressing mGPC3 (Fig. 3D). Furthermore, confirming whether the mGPC3-1 peptide-specific CTL line killed cancer cells presenting the mGPC3-1 peptide is important, thus a cytotoxicity assay was performed (Fig. 3B).

Mice with a B6 background that spontaneously develop liver cancer have been reported (19,20). These mice enable investigations as to whether a peptide vaccine for GPC3 has a preventive capability. Recently, the STAM mice C57BL/6N-NASH was established as a non-alcoholic-steatohepatitis (NASH) mouse model by Stelic Institute & Co. STAM mice C57BL/6N-NASH are drug-treated B6 mice and liver cancer occurs spontaneously and early in NASH mice. Therefore, this mouse is an attractive model for studying the preventive effects of a cancer vaccine. We showed that mGPC3-1 peptide-specific CTL could be induced in STAM mice C57BL/6N-NASH (Fig. 4E). Simultaneously, we established a liver cancer cell line derived from STAM mice C57BL/6N-NASH and observed the cancer cell line expressed mGPC3 (data not shown).

However, the GPC3 peptide vaccine did not prevent the occurrence of liver cancer in STAM mice C57BL/6N-NASH (data not shown). Therefore, further research to develop strong GPC3-specific immunotherapies or combinational approaches in an appropriate mouse model is needed. Identification of an H2-K^b^ or H2-D^b^ restricted, GPC3-derived peptide is the first step. The established cell line from STAM mice C57BL/6N-NASH, which show GPC3 expression, may help us to develop a new mouse model system for a GPC3-targeted therapy.

In conclusion, mGPC3-1_127–136_ AMFKNNYPSL was identified as an H2-K^b^ or H2-D^b^ restricted, GPC3-derived CTL most-inducible epitope peptide and mGPC3-1 peptide-specific CTL can kill RMA-S pulsed with the mGPC3-1 peptide. Furthermore, we established an mGPC3-1-specific CTL-inducible model in B6 mice using an mGPC3-1 peptide vaccine.

## Figures and Tables

**Table I:** Synthetic peptides predicted to bind with H2-K^b^.

	Peptide sequence (position)	Binding score^a^
mGPC3-1	AMFKNNYPSL (127–136)	52.8
mGPC3-2	SLFPVIYTQM (172–181)	44
mGPC3-3	LFPVIYTQM (173–181)	66
mGPC3-4	KSFINFYSAL (395–404)	40

a Binding scores were estimated by using BIMAS software (http://www-bimas.cit.nih.gov/molbio/hla_bind/).

**Table II:** Synthetic peptides predicted to bind with H2-D^b^.

	Peptide sequence (position)	Binding score^a^
mGPC3-5	LTARLNMEQL (82–91)	200
mGPC3-1	AMFKNNYPSL (127–136)	343.2
mGPC3-6	LGSDINVDDM (156–165)	260
mGPC3-7	QYVQKNGGKL (331–340)	720
mGPC3-8	YVQKNGGKL (332–340)	240
mGPC3-9	DTLCWNGQEL (418–127)	600
mGPC3-10	RNGMKNQFNL (437–446)	200
mGPC3-11	MKNQFNLHEL (440–449)	288

a Binding scores were estimated by using BIMAS software (http://www-bimas.cit.nih.gov/molbio/hla_bind/).
